# Unicompartmental knee arthroplasty is superior to high tibial osteotomy for the treatment of medial unicompartmental osteoarthritis: A systematic review and meta-analysis

**DOI:** 10.1097/MD.0000000000029576

**Published:** 2022-07-29

**Authors:** Linke Huang, Yinglong Xu, Linhua Wei, Guangzhi Yuan, Weiwei Chen, Shiyao Gao, Wei Liu, Zhen Tan, Jinmin Zhao

**Affiliations:** a Department of Orthopaedics, The Second Affiliated Hospital of Guangxi Medical University, Nanning, GuangxiChina; b Department of Orthopaedics, The First Affiliated Hospital of Guangxi Medical University, Nanning, GuangxiChina; c Guangxi Key Laboratory of Regenerative Medicine, Guangxi Medical University, Nanning, GuangxiChina; d Department of Orthopaedics, The Fifth Affiliated Hospital of Guangxi Medical University, Nanning, GuangxiChina; e The Affiliated Nanning Infectious Disease Hospital of Guangxi Medical University, The Fourth People’s Hospital of Nanning, Nanning, GuangxiChina.

**Keywords:** high tibial osteotomy, medial unicompartmental osteoarthritis, meta-analysis, unicompartmental knee arthroplasty

## Abstract

**Background:**

Unicompartmental knee arthroplasty (UKA) and high tibial osteotomy (HTO) are widely used for the treatment of medial unicompartmental knee osteoarthritis (OA). However, the best approach remains controversial. This study aimed to present a systematic review and a meta-analysis to directly compare the clinical outcomes between HTO and UKA. We hypothesized that the clinical outcomes after UKA and HTO would be similar.

**Methods:**

Electronic databases (Web of Science, PubMed, Embase, CENTRAL, and Biosis Preview) were searched for related studies published before November 30, 2021. Retrospective and prospective studies that directly compared the postoperative outcomes between UKA and HTO were included. Odds ratio (ORs) and 95% confidence interval (CIs) for complications, revision to total knee arthroplasty (TKA), and weighted mean difference (MD) and 95% CIs in range of motion (ROM), pain, walking speed and function score were evaluated. Two reviewers independently assessed the quality of the studies. Subgroup and sensitivity analyses were performed to explore the heterogeneity.

**Results:**

Twenty-three retrospective and 6 prospective studies were included. A total of 3004 patients (3084 knees) were evaluated for comparison. Complications (OR, 4.88, 95% CI: 2.92–6.86) were significantly greater in the HTO group than in the UKA group. Postoperative function scores including Lysholm score (MD, −2.78, 95% CI: −5.37 to −0.18) and Hospital for Special Surgery (HSS) score (MD, −2.80, 95% CI: −5.39 to −0.20) were significantly lower in the HTO group than the UKA group. The postoperative ROM was similar between HTO and mobile-bearing UKA (MD, −3.78, 95% CI: −15.78 to 8.22). However, no significant differences were observed between the HTO and UKA group in terms of postoperative pain, walking speed, and revision to TKA.

**Conclusions:**

UKA is superior to HTO in minimizing complications and enhancing postoperative function scores. Mobile-bearing UKA has a similar ROM compared with HTO. Both HTO and UKA provide satisfactory clinical outcomes in terms of walking speed, relieving pain, and revision to TKA. UKA appears to be more suitable for the elderly, and both mobile-bearing UKA and HTO are viable surgical options for younger active individuals.

## 1. Introduction

Osteoarthritis (OA) is a major cause of impairment that affects more people than any other joint disease. Knee joints are most frequently afflicted by OA.^[1]^ It has been reported that arthritic change is mostly found in the medial compartment in 10%–29.5% of all cases.^[2,3]^ For mild medial unicompartmental knee OA (Kellgren–Lawrence grade [K–L grade] I), conservative therapy, including functional training, physical therapy, intra-articular injection, and oral medicines, can relieve pain and improve quality of life.^[4]^ However, for more serious medial unicompartmental knee OA (K–L grade II–IV), operations may obtain more ideal results.^[5,6]^

High tibial osteotomy (HTO) and unicompartmental knee arthroplasty (UKA) are widely used in the treatment of medial unicompartmental knee OA.^[7,8]^ HTO and UKA differ in terms of concept. HTO is designed to increase the life span of articular cartilage by unloading and redistributing the mechanical forces over the unaffected compartment, while UKA is introduced to resurface the degenerative compartment and preserve the unaffected compartment. Several studies compared the outcomes of HTO and UKA,^[9–14]^ and the predominance of either procedure is ambiguous. Generally, HTO is regarded as a better option for younger and physically active patients, and those treated with UKA benefit from a faster recovery process.^[15]^ Nevertheless, with improvements in operative techniques and the quality of the implants, UKA could also obtain a good response to its activity.^[5,7]^ It is difficult for surgeons to decide which one is more suitable for patients with medial unicompartmental OA.

This meta-analysis aimed to directly compare the clinical outcomes of HTO and UKA involving complications, revisions to TKA, range of motion (ROM), walking speed, pain, and function score in the treatment of medial unicompartmental knee OA by reviewing relevant studies. We hypothesized that the clinical outcomes after UKA and HTO would be similar.

## 2. Methods

### 2.1. Search strategy

The study was conducted following the Preferred Reporting Items for Systematic Reviews and Meta-Analysis (PRISMA) statement. All coauthors determined the research protocol before starting the bibliographic searches. The study protocol was recorded in the PROSPERO International Prospective Register of Systematic Reviews, Number CRD42020165829. The electronic databases PubMed, Embase, Web of Science, CENTRAL (using Ovid platform), and Biosis Preview (using Ovid platform) were searched for related studies until before November 30, 2021, without language restrictions. To maximize the search sensitivity and specificity, the search strategy for the 5 databases followed the combination of Medical Subject Headings with expressions. Ethical approval was not required, as this study utilized published data.

### 2.2. Selection criteria

Two reviewers independently evaluated the search outcomes for inclusion in this systematic review byinspecting abstracts, titles, or complete texts. Discordance between the 2 investigators was settled through consensus or discussion with a third researcher. All prospective and retrospective controlled studies that met the inclusion criteria were included. The inclusion criteria were as follows: (1) the study was a retrospective or prospective controlled trial; (2) the subjects were patients with medial unicompartmental OA; (3) the study directly compared HTO with UKA; and (4) the study included at least one outcome, such as pain, complications, functions, and revision to total knee arthroplasty. The exclusion criteria were as follows: (1) case series; (2) registration study; (3) cost-effectiveness study; (4) study indirectly comparing HTO with UKA; (5) no valid data could be extracted from a published study; (6) cadaver or animal studies; (7) studies that included patients with traumatic arthritis or rheumatoid arthritis.

### 2.3. Data extraction

Data were independently extracted from the selected studies by 2 researchers. The extracted data included study name, date, study design, participant demographics, baseline characteristics, and outcomes. The study authors were contacted by email to clarify unclear data. Data were recorded using Microsoft Excel and Word software. Outcomes included surgical complications (e.g. infection), pain score, functional score (e.g., Lysholm score), range of motion (ROM), walking speed, and revision to TKA at the last follow-up.

### 2.4. Study quality evaluation

The study quality was independently evaluated by 2 reviewers. The Newcastle–Ottawa scale was employed to classify the bias risk of non-randomized comparative studies corresponding to each study design (cohort or case-control)^[16]^ and the Cochrane Risk of Bias tool was employed to evaluate randomized control trials.^[17]^ Discordance between the 2 investigators was settled through consensus or discussion with a third researcher.

### 2.5. Statistical analysis

The RevMan 5.3 software for Windows was employed to perform statistical analysis. Dichotomous data were evaluated using the odds ratio (OR) and associated 95% confidence interval (CI) through the Mantel-Haenszel (M-H) Method. The weighted mean difference (MD) and corresponding 95% CI values were used to define the continuous data value through the Inverse Variance Method. *I*^2^ tests and *P* values for the Cochrane Q tests were used to evaluate heterogeneity. When *I*^2^ value was < 50% and *P* value was > .1, a fixed-effects model was employed for the meta-analysis; otherwise, a random-effects model was selected. Subgroup and sensitivity analyses were performed to explore potential sources of heterogeneity. A funnel plot was used to examine the publication bias.

## 3. Results

### 3.1. Literature screening process and results

**Figure 1.**
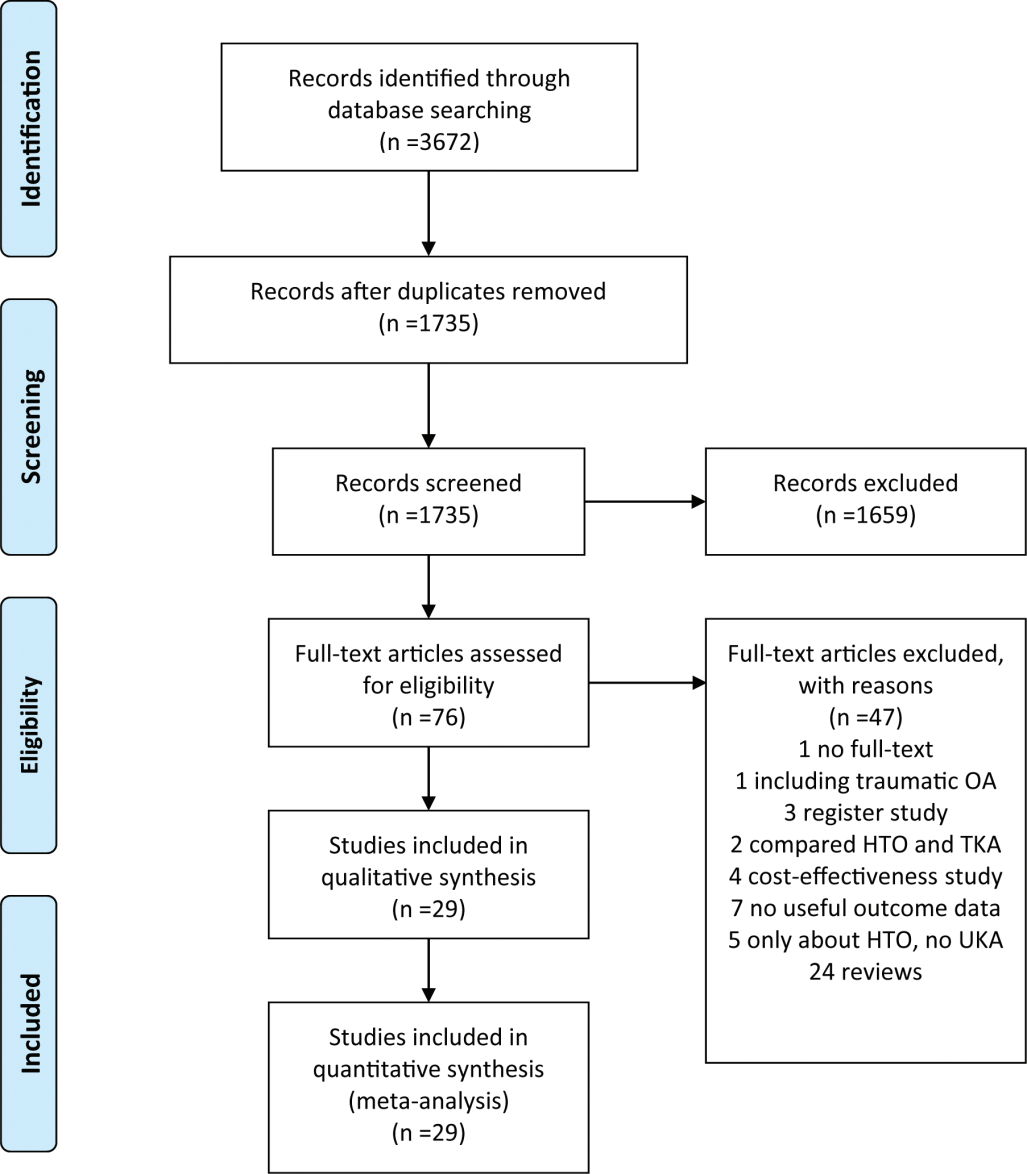
Preferred reporting items for systemic reviews and meta-analyses (PRISMA) flow diagram of literature selection.

A total of 3672 published manuscripts were retrieved from 5 electronic database searches. 1937 duplicates were removed and 1659 records were excluded after screening the titles or abstracts. Because there was no useful information or other reasons, an additional 47 studies were removed based on abstracts or complete articles. Eventually, 29 studies that met the inclusion criteria were included, 6 of them were prospective randomized trials and 23 were retrospective comparative studies.^[9–11,13,14,18–41]^ The literature screening process and the results are shown in Fig. 1.

### 3.2. Study characteristics and quality

**Figure 2.**
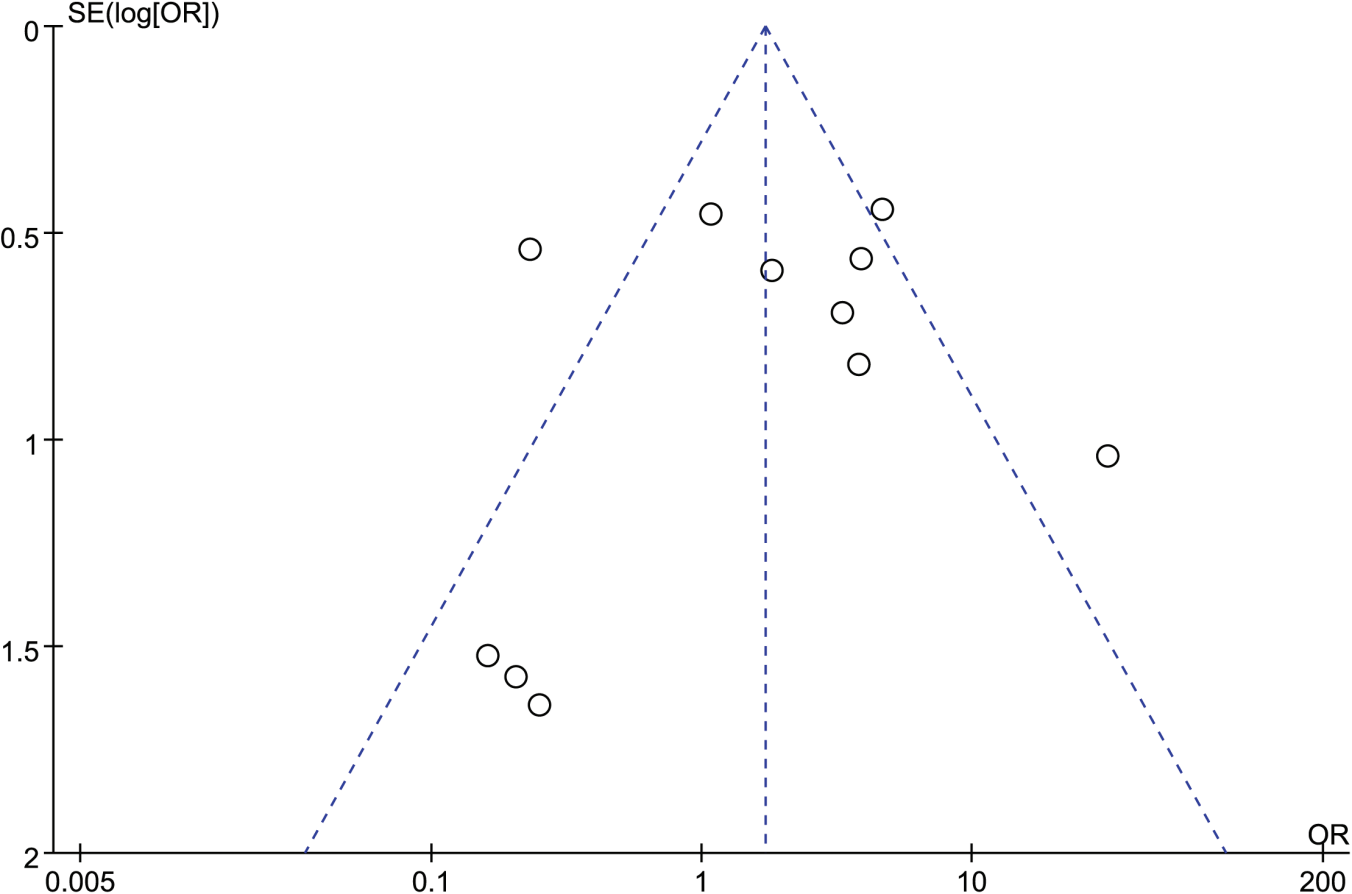
Funnel plot of revision to TKA to assess publication bias. OR = odds ratio; SE = standard error.

A total of 1388 patients/1412 knees were treated with HTO and 1616 patients/1672 knees were treated with UKA for medial compartmental knee OA.^[9–11,13,14,18–41]^ The minimum mean follow-up times for HTO and UKA were 3.6 months and 3.7 months,^[11]^ respectively. The maximum follow-up time for HTO and UKA was 17 years for both.^[32]^ The HTO type has been reported as opening-wedge HTO (OWHTO)^[9–11,18,19,21–24,26,27,29,35–41]^ and closing-wedge HTO (CWHTO)^[13,14,20,30–34]^ in eighteen and 8 studies, respectively. One study included 38 OWHTO and 19 CWHTO,^[25]^ and 1 study included 57 OWHTO and 36 Demo-HTO.^[28]^ UKA protheses have been reported as fixed-bearing^[9,11,13,14,19,20,23,25,26,29–32]^ and mobile-bearing UKA^[10,21,22,24,27,28,33–36,38,39]^ in 13 and 12 studies, respectively, but 4 studies did not mention the UKA type.^[18,37,40,41]^ Tables 1,2 show the details of the included studies, and Tables 3,4 show their quality. Revision to TKA, which was the most cited result, was employed to obtain a funnel plot analysis of publication bias. The asymmetric features of the funnel plot suggest a certain publication bias (Fig. 2).

**Table 1:** Summary of characteristics, patient demographic details for each study.

Study	Design	HTO type/UKA model		Number of operation knees	Age (y)	Female/Male	BMI (kg/m^2^)	OA severtity grade		Follow-up
Liu 2021	Retrospective	OWHTO/MB	HTO	48	59.5 ± 3.5	32/16	28.1 ± 1.8	K-L grade 2/3		3.3 Y
			UKA	49	61.2 ± 2.8	31/18	27.3 ± 2.1			3.5 Y
Watanabe 2021	Retrospective	OWHTO/UKA	HTO	48(46 patients)	61.3 ± 9.8	NC	26.1 ± 3.8	K–L grade 2/3		22.0 M
			UKA	48(44 patients)	73.8 ± 5.2		24.1 ± 2.8			22.5 M
Rodkey 2021	Retrospective	OWHTO/UKA	HTO	113	40	12/101	29.8	NC		5.3 Y
			UKA	270	48	80/190	31.0			6.3 Y
Lin 2021	Retrospective	OWHTO/MB	HTO	53	56.0 ± 10.2	40/13	26.3 ± 3.2	K–L grade 2/3		More than 1 y
			UKA	61	61.4 ± 4.7	46/15	26.0 ± 3.1			
Jin 2021	Retrospective	OWHTO/FB	HTO	67	64.1 ± 4.0	67/0	25.5 ± 3.1	K–L grade 3/4	16/51	More than 1 y
			UKA	67	63.1 ± 4.9	65/2	25.5 ± 2.8		15/52	
Zhang 2020	Retrospective	OWHTO/UKA	HTO	109	51.8 ± 6.9	86/23	26.4 ± 3.6	NC		40.2 ± 13.5 M
			UKA	83	53.7 ± 5.2	66/17	27.7 ± 4.1			39.3 ± 11.2 M
Hou 2020	Retrospective	OWHTO/MB	HTO	30	NC	NC	NC	NC		NC
			UKA	30						
Chen 2020	Retrospective	OWHTO/MB	HTO	18	56.1 ± 6.4	13/5	NC	K–L grade 2/3		1.0–2.8 Y
			UKA	20	56.1 ± 6.5	14/6				1.0–3.2 Y
Jacquet 2020	Retrospective	OWHTO/FB	HTO	50	49.3 ± 3.9	22/28	26.6 ± 2.6	NC		3.7 ± 1.6 Y
			UKA	50	50.8 ± 4.4	21/29	27.1 ± 3.1			3.9 ± 1.8 Y
Song 2019	Retrospective	CWHTO/FB	HTO	60	59.7 ± 4.1	51/9	25.1 ± 3.6	K–L grade 3/4	50/10	10.7 ± 5.7 Y
			UKA	50	60.8 ± 3.9	43/7	25.3 ± 3.4		34/36	12.0 ± 7.1 Y
Koh 2019	Retrospective	OWHTO/MB	HTO	123	56.1 ± 5.6	104/19	25.9 ± 3.2	AH grade <2/≥2	97/26	2 Y
			UKA	118	60.8 ± 4.7	98/20	25.8 ± 2.9		37/81	2 Y
Kim 2019	Prospective	OWHTO/MB	HTO	49	56.1 ± 6.2	43/6	26.6 ± 9.2	K–L grade 2/3/4	9/28/12	2 Y
			UKA	42	63.6 ± 5.5	35/7	25.3 ± 2.4		0/23/19	2 Y
Ryu 2018	Retrospective	OWHTO/FB	HTO	23	57.6 ± 6.4	22/1	27.7 ± 2.9	NC		40.0 ± 19.4 M
			UKA	22	60.5 ± 3.4	19/3	25.4 ± 3.6			33.1 ± 8.7 M
Cho 2018	Retrospective	OWHTO/ MB	HTO	20(17 patients)	58.4 ± 5.5	12/8	26.5 ± 2.5	AH grade 2/3	18/2	48.4 ± 14.3 M
			UKA	20(17 patients)	67.9 ± 9.0	19/1	26.1 ± 2.6		13/7	39.7 ± 14.0 M
Zhao 2017	Prospective	OWHTO/UKA	HTO	36	53.91 ± 7.35	33/3	NC	K–L grade 2/3	10/26	2 Y
			UKA	36	52.47 ± 8.41	31/5			11/25	2 Y
Maxwell 2017	Retrospective	OWHTO/MB	HTO	75	Under 55	NC	NC	NC		8.1 Y
			UKA	95	Under 55					6.1 Y
Krych 2017	Retrospective	OWHTO, CWHTO/MB	HTO	57	42.7	16/41	31.8	NC		>2 Y
			UKA	183	49.2	95/88	32.4			>2 Y
Jeon 2017	Retrospective	OWHTO/FB	HTO	26	56.8	22/4	26.6	NC		34.7 M
			UKA	21	60.7	17/4	26.1			34.7 M
Petersen 2016	Retrospective	OWHTO/ MB	HTO	23	58.9 ± 2.8	9/14	23	AH grade 1/2/3	14/9/0	>5 Y
			UKA	25	60.7 ± 2.5	16/9	25		1/20/5	>5 Y
Tuncay 2015	Retrospective	OWHTO, Demo-HTO/MB	HTO	93(88 patients)	51.7, 53.5	70/18	NC	NC		40.4 M, 30.7 M
			UKA	109(94patients)	58.7	79/15				42.5 M
Yim 2013	Retrospective	OWHTO/FB	HTO	58	58.3 ± 5.4	51/7	NC	NC		3.6 ± 0.4 Y
			UKA	50	60.3 ± 4.5	48/2				3.7 ± 0.4 Y
Takeuchi 2010	Retrospective	OWHTO/FB	HTO	27(24 patients)	67 ± 7	18/6	NC	AH grade 2/3/4/5	11/14/2/0	61 ± 10 M
			UKA	30(18 patients)	77 ± 4	14/4			4/17/8/1	84 ± 4 M
Borjesson 2005	Prospective	CWHTO/FB	HTO	18	63 ± 3	8/10	NC	AH grade 1/2/3	4/7/7	5 Y
			UKA	22	63 ± 4	11/11			7/6/9	5 Y
Stukenborg 2001	Prospective	CWHTO/FB	HTO	32(32 patients)	67(60–79)	13/19	NC	AH grade 1/2/3/4/5	18/7/1/5/1	7–10 Y
			UKA	30(28 patients)	67(60–80)	22/6			11/9/4/6/0	7–10 Y
Weale 1994	Retrospective	CWHTO/FB	HTO	49(45 patients)	74	NC	NC	NC		12–17 Y
			UKA	42(34 patients)	80					12–17 Y
Weidenhielm 1992	Prospective	CWHTO/FB	HTO	25	63	14/11	NC	AH grade 1/2/3	4/14/10	1 Y
			UKA	28	63	14/14			3/10/12	1 Y
Ivarsson 1991	Retrospective	CWHTO/FB	HTO	10	62 ± 4	6/4	NC	AH grade 1/2/3	4/5/1	6 M
			UKA	10	64 ± 5	6/4			2/4/4	12 M
Jefferson 1989	Prospective	CWHTO/MB	HTO	23(20 patients)	57(31–73)	NC	NC	NC		NC
			UKA	19(15 patients)	64(55–74)					NC
Broughton 1986	Retrospective	CWHTO/FB	HTO	49(45 patients)	63	NC	NC	K–L grade	3.3	7.8 ± 1.5 Y
			UKA	42(34 patients)	71				3.2	5.8 ± 1.2 Y

**Table 2:** Summary of clinical outcomes for each study.

Study		Complication	Revision	Pain (VAS)	ROM	Function score	Walking speed
Liu 2021	HTO	0	0	NC	134.2 ± 2.7	93.5 ± 5.0 (Lysholm)	NC
	UKA	0	0		133.4 ± 3.1	93.9 ± 4.1 (Lysholm)	
Watanabe 2021	HTO	NC	NC	NC	131.9 ± 8.2	80.8 ± 17.5 (Lysholm)	NC
	UKA				129.5 ± 12.3	82.4 ± 16.4 (Lysholm)	
Rodkey 2021	HTO	24	4	NC	NC	NC	NC
	UKA	6	37				
Lin 2021	HTO	2	NC	NC	NC	86.58 ± 4.28 (HSS)	NC
	UKA	1				87.72 ± 2.80 (HSS)	
Jin 2021	HTO	1	7	NC	138.1 ± 3.7	86.6 ± 11.5 (HSS)	NC
	UKA	0	2		135.7 ± 10.0	88.8 ± 13.2 (HSS)	
Zhang 2021	HTO	0	0	0.5 ± 0.7	NC	90.6 ± 8.7 (HSS)	NC
	UKA	2	1	0.5 ± 0.6		91.7 ± 7.2 (HSS)	
Hou 2020	HTO	NC	NC	1.8 ± 0.9	121.1 ± 2.7	81.9 ± 14.3 (HSS)	NC
	UKA			1.9 ± 0.8	135.2 ± 1.6	82.6 ± 12.9 (HSS)	
Chen 2020	HTO	0	0	NC	NC	90.6 ± 2.0(Lysholm)	NC
	UKA	0	0			91.5 ± 1.7(Lysholm)	
Jacquet 2020	HTO	5	0	1.0 ± 0.5	NC	61 ± 7(KSS)	NC
	UKA	0	0	0.9 ± 0.4		60 ± 9(KSS)	
Song 2019	HTO	NC	14	NC	135.3 ± 12.3	73.9 ± 15.3(KSS) 30.6 ± 16.6(WOMAC)	NC
	UKA		11		126.8 ± 13.3	71.0 ± 10.5(KSS) 32.9 ± 10.2(WOMAC)	
Koh 2019	HTO	0	NC	2.6 ± 1.3	NC	24.2 ± 11.4(WOMAC)	NC
	UKA	0		2.2 ± 1.6		13.9 ± 6.4(WOMAC)	
Kim 2019	HTO	NC	NC	4.7	NC	NC	NC
	UKA			5.2			
Ryu 2018	HTO	0	NC	2.2 ± 1.2	NC	16.5 ± 17.5(WOMAC)	NC
						87.4 ± 12.0(Lysholm)	
	UKA	0		1.5 ± 1.7		14.9 ± 16.9(WOMAC)	
						89.2 ± 10.2(Lysholm)	
Cho 2018	HTO	0	NC	NC	149.4 ± 9.4	95.1 ± 7.6(KSS)	NC
	UKA	1			146.8 ± 12.7	96.3 ± 8.5(KSS)	
Zhao 2017	HTO	NC	NC	3.54 ± 0.50	126.13 ± 1.45	82.76 ± 8.13(HSS)	NC
	UKA			2.45 ± 0.47	128.94 ± 1.37	93.09 ± 8.69(HSS)	
Maxwell 2017	HTO	18	19	NC	NC	21 (FJS)	NC
	UKA	3	1			67(FJS)	
Krych 2017	HTO	NC	13	NC	NC	80.2 ± 11.8(Lysholm)	NC
	UKA		11			90.0 ± 11.0(Lysholm)	
Jeon 2017	HTO	2	NC	3.3077 ± 2.61119	NC	50.1727 ± 17.12898(IKDC)	NC
	UKA	1		2.2381 ± 2.16575		56.8667 ± 15.70697(IKDC)	
Petersen 2016	HTO	2	NC	NC	NC	7/10/2/4(HSS, Excellent/Good/Fair/Poor)	NC
	UKA	1				14/7/2/2(HSS, Excellent/Good/Fair/Poor)	
Tuncay 2015	HTO	6	0	NC	NC	OWHTO 83.95(HSS)	NC
						Demo-HTO 83.51(HSS)	
	UKA	1	3			90.00(HSS)	
Yim 2013	HTO	3	NC	NC	138.8 ± 4.7	89.6 ± 8.7(Lysholm)	NC
	UKA	2			130.0 ± 8.8	90.3 ± 7.7(Lysholm)	
Takeuchi 2010	HTO	2	0	NC	146 ± 5.9	89 ± 7.6(KSS)	NC
	UKA	1	2		127 ± 16	88 ± 7.7(KSS)	
Borjesson 2005	HTO	NC	NC	0 (0–2) (Borg-scale)	121	37(36–39) (BOA)	NC
	UKA			0 (0–2) (Borg-scale)	121	37(31–39) (BOA)	
Stukenborg 2001	HTO	9	10	NC	117(85–135)	76(29–100) (KSS)	NC
	UKA	2	6		103(35–140)	74(31–94) (KSS)	
Weale 1994	HTO	NC	17	9/21 (no/mild)	NC	31(BKS)	NC
	UKA		5	12/15 (no/mild)		34(BKS)	
Weidenhielm 1992	HTO	2	NC	25 (No pain)	NC	37 ± 2(BOA)	1.29 ± 0.16
	UKA	1		28 (No pain)		38 ± 2(BOA)	1.3 ± 0.18
Ivarsson 1991	HTO	NC	NC	6.3 ± 2.1 (100 mm Analogous)	121 ± 11	78 ± 19(Lysholm)	1.35 ± 0.42
	UKA			4.1 ± 2.9 (100 mm Analogous)	112 ± 13	91 ± 11(Lysholm)	1.20 ± 0.24
Jefferson 1989	HTO	NC	NC	NC	NC	NC	1.02 ± 0.19
	UKA						0.99 ± 0.21
Broughton 1986	HTO	17	10	23 (No or mild)	NC	21 (BKS good number)	NC
	UKA	5	3	34 (No or mild)		32 (BKS good number)	

**Table 3:** Risk of bias assessment using the Newcastle–Ottawa scale for cohort studies included in the systematic review and meta-analysis.

	Selection				Comparability	Exposure		
Study	Representativeness of the exposed cohort (*)	Selection of the nonexposed cohort (*)	Ascertainment of exposure (*)	Demonstration that outcome of interest was not present at start of study (*)	Comparability of cohorts on the basis of the design or analysis (**)	Assessment of outcome (*)	Was follow-up long enough for outcomes to occur (*)	Adequacy of follow up of cohorts (*)
Liu 2021	*	*	*	*	*	*	*	*
Watanabe 2021	*	*	*	*	*	*	*	*
Rodkey 2021	*	*	*	*	*	*	*	*
Lin 2021	*	*	*	*	*	*	*	*
Jin 2021	*	*	*	*	*	*	*	*
Zhang 2020	*	*	*	*	*	*	*	*
Hou 2020	*	*	*	*		*	*	*
Chen 2020	*	*	*	*	*	*	*	*
Jacquet 2020	*	*	*	*	**	*	*	*
Song 2019	*	*	*	*	**	*	*	*
Koh 2019	*	*	*	*		*	*	*
Ryu 2018	*	*	*	*	*	*	*	*
Cho 2018	*	*	*	*		*	*	*
Maxwell 2017	*	*	*	*		*	*	*
Krych 2017	*	*	*	*		*	*	*
Jeon 2017	*	*	*	*	**	*	*	*
Petersen 2016	*	*	*	*		*	*	*
Tuncay 2015	*	*	*	*	*	*	*	*
Yim 2013	*	*	*	*	**	*	*	*
Takeuchi 2010	*	*	*	*	*	*	*	*
Weale 1994	*	*	*	*	**	*	*	*
Ivarsson 1991	*	*	*	*	*	*		*
Broughton 1986	*	*	*	*	*	*	*	*

**Table 4:** Risk of bias assessment using the Cochrane risk of bias tool for RCTs included in the systematic review and meta-analysis.

Study	Random sequence generation	Allocation concealment	Blinding of participants and personnel	Blinding of outcome assessment	Incomplete outcome data	Selective reporting	Other bias
Kim 2019	High risk	High risk	Low risk	Low risk	Low risk	Low risk	Unclear risk
Zhao 2017	Unclear risk	Unclear risk	Low risk	Low risk	Low risk	Low risk	Unclear risk
Borjesson 2005	Unclear risk	Unclear risk	Low risk	Low risk	Low risk	Low risk	Unclear risk
Stukenborg 2001	Low risk	Low risk	Low risk	Low risk	Low risk	Low risk	Unclear risk
Weidenhielm 1992	Unclear risk	Unclear risk	Low risk	Low risk	Low risk	Low risk	Unclear risk
Jefferson 1989	High risk	High risk	Low risk	Low risk	Low risk	Low risk	Unclear risk

### 3.4. Outcomes

#### 3.4.1. Complications.

**Figure 3.**
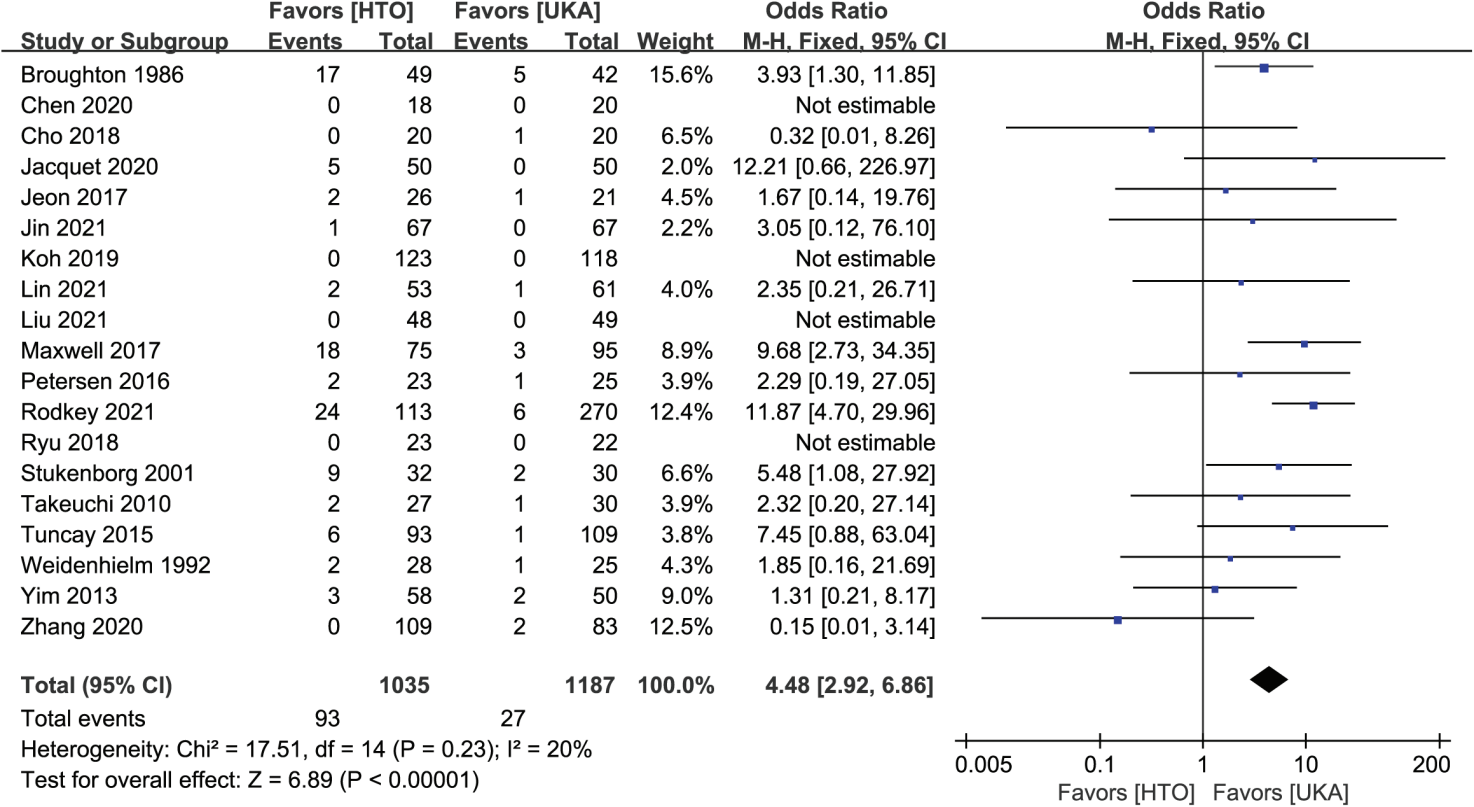
Forest plots for comparison of complications between HTO and UKA patients. CI = confidence interval; HTO = high tibial osteotomy; UKA = unicompartmental knee arthroplasty.

Complications included hematoma, vessel and nerve injury, infection, deep venous thrombosis or pulmonary embolism, nonunion, dislocation, failure of fixation, and bedsores. Nineteen studies reported procedure-related complications.^[9–11,13,14,19,21,23,24,26–29,31,36–39,41]^ Complications occurred at a significantly higher rate in the HTO group than in the UKA group (OR, 4.88, 95% CI: 2.92–6.86, *I^2^* = 20%, *P* < .00001; Fig. 3).

#### 3.4.2. Revision to TKA.

**Figure 4.**
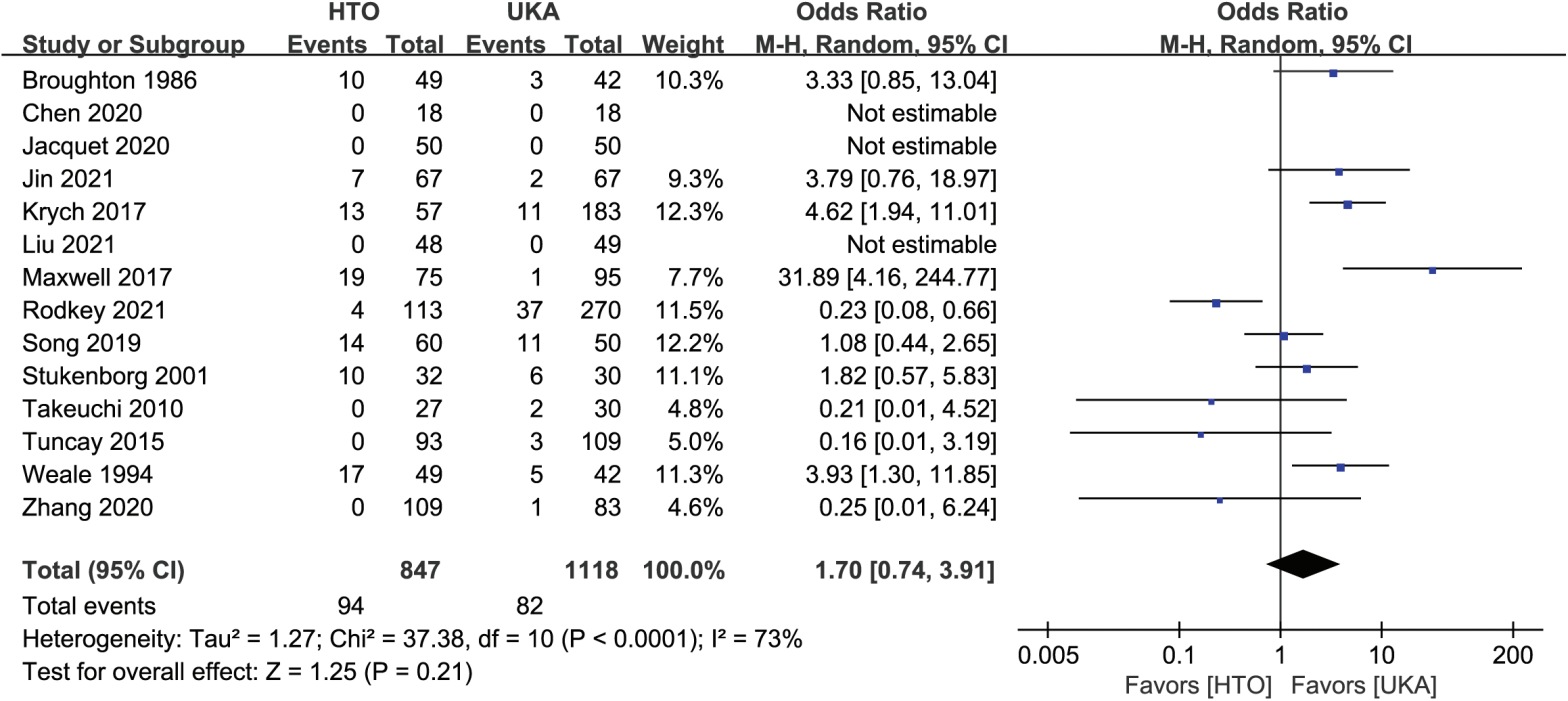
Forest plots for revision to TKA comparison between HTO and UKA patients. CI = confidence interval; HTO = high tibial osteotomy; UKA = unicompartmental knee arthroplasty.

Fourteen studies with 1965 patients reported 176 subjects who underwent revision to TKA.^[9,10,14,19,20,25,28,29,31,32,37–39,41]^ The pooled data showed that the OR for the revision rate was 1.70 (95% CI: 0.74–3.91, *I^2^* = 73%, *P* = .21; Fig. 4), and there was no significant difference between the 2 groups. Sensitivity analysis was performed to explore potential sources of heterogeneity. After excluding the study of Rodkey 2021, the OR for the revision rate was 2.35 (95% CI: 1.16–4.77, *I^2^* = 56%, *P* = 0.02). HTO had a greater revision rate than UKA.

#### 3.4.3. Range of motion.

**Figure 5.**
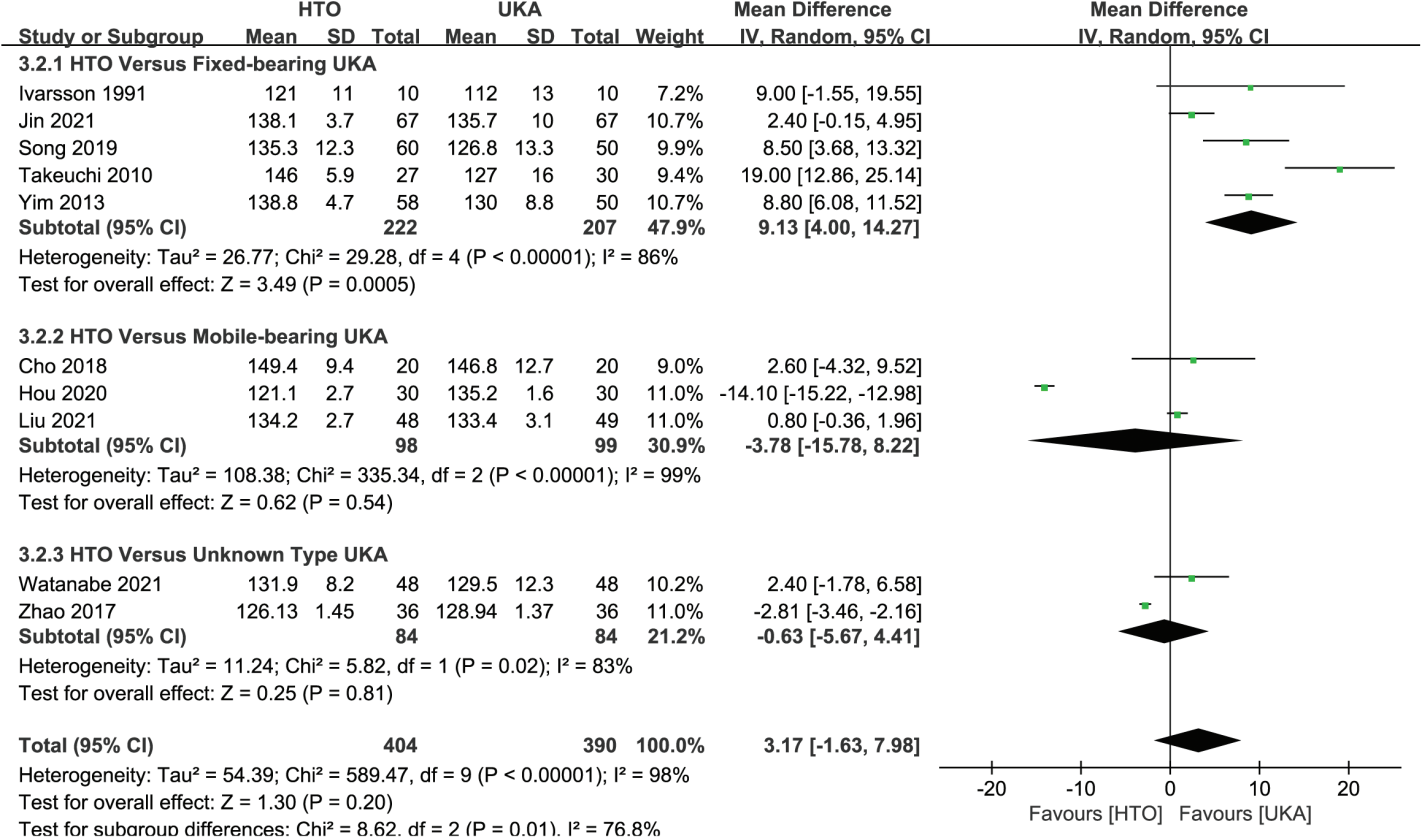
Forest plots for ROM comparison between HTO and UKA patients. CI = confidence interval; ROM = range of motion; HTO = high tibial osteotomy; UKA = unicompartmental knee arthroplasty.

Ten studies^[9,11,18,20,24,29,33,35,39,40]^ compared ROM between HTO and UKA. The pooled data suggested that the difference in ROM between the HTO and UKA groups was not statistically significant (MD, 3.17, 95% CI: −1.63 to 7.98, *I^2^* = 98%, *P* = .20; Fig. 5). In subgroup analysis, the HTO group showed a greater motion range than the fixed-bearing UKA group (MD, 9.13, 95% CI: 4.00–14.27, *I^2^* = 68%, *P* = .0005; Fig. 5). However, no significant difference was observed in ROM between HTO and mobile-bearing UKA groups (MD −3.78, 95% CI: −15.78 to 8.22, *I^2^* = 99%, *P* = .54; Fig. 5) and unknown type UKA groups (MD, −0.63, 95% CI: −5.67 to 4.41, *I^2^* = 83%, *P* = .81; Fig. 5).

#### 3.4.4. Postoperative pain.

**Figure 6.**
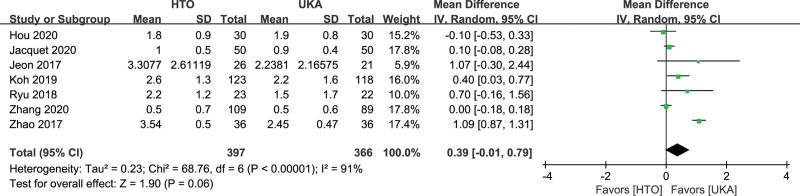
Forest plots for postoperative pain comparison between HTO and UKA patients. CI = confidence interval; HTO = high tibial osteotomy; UKA = unicompartmental knee arthroplasty.

Thirteen studies reported postoperative pain.^[13,14,18,19,21–23,26,30,32,33,35,41]^ Different assessment systems have been used to assess pain. Based on the available data, 7 studies^[18,19,21,23,26,35,41]^ included information on postoperative pain, which was assessed by the visual analogue scale (VAS).^[42]^ The present analysis showed no statistically significant difference between the 2 groups (MD, 0.39, 95% CI: −0.01 to 0.79, *I^2^* = 91%, *P* = .06; Fig. 6).

#### 3.4.5. Walking speed.

**Figure 7.**
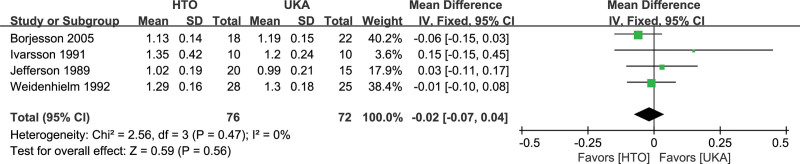
Forest plots for walking speed comparison between HTO and UKA patients. CI = confidence interval; HTO = high tibial osteotomy; UKA = unicompartmental knee arthroplasty.

No significant differences were found in walking speed between the HTO and UKA groups (MD, −0.02, 95% CI: −0.07 to 0.04, *I^2^* = 0%, *P* = .56; Fig. 7), as reported in 4 studies^[13,30,33,34]^ involving 148 knees.

#### 3.4.6. Function score.

**Figure 8.**
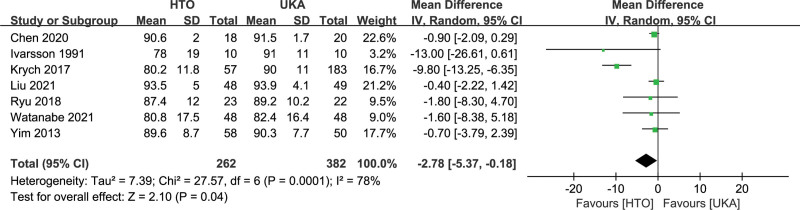
Forest plots for Lysholm score comparison between HTO and UKA patients. CI = confidence interval; HTO = high tibial osteotomy; UKA = unicompartmental knee arthroplasty.

There are several different scoring systems to compare the postoperative functional outcomes between the 2 groups.^[9–11,13,14,18–33,35,36,38–41]^ However, only a few studies provided the mean and standard deviation (SD) of the same scoring system used in our meta-analysis. Seven studies^[11,23,25,33,38–40]^ involving 644 patients reported a lower Lysholm score in the HTO group than in the UKA group (MD −2.78, 95% CI: −5.37 to −0.18, *I^2^* = 78%, *P* =.04; Fig. 8). The Hospital for Special Surgery (HSS) score was estimated in 6 studies.^[9,18,23,35,36,41]^ The HTO group had worse HSS than the UKA group (MD, −2.80, 95% CI: −5.39 to −0.20, *I^2^* = 75%, *P* = .03). Five studies^[9,19,20,24,29]^ used the Knee Society Score (KSS) to assess postoperative knee function. The results showed no significant difference between the HTO and UKA groups (MD, −0.26, 95% CI: −1.94 to 1.41, *I^2^* = 33%, *P* = .76). Four studies^[9,20,21,23]^ used the Western Ontario and McMaster Universities Osteoarthritis Index (WOMAC). The meta-analysis of WOMAC results showed no differences between the 2 groups (MD, 4.33, 95% CI: −1.91 to 10.56, *I^2^* = 86%, *P* = 0.17). The Tegner score was also similar between the 2 groups (MD, −0.35, 95% CI: −0.90 to 0.20, *I^2^* = 89%, *P* = .21).

## 4. Discussion

The main findings of this meta-analysis were that UKA was superior to HTO in terms of complications and postoperative function scores (Lysholm and HSS scores). Subgroup analysis revealed that mobile-bearing UKA had a similar ROM to HTO, whereas fixed-bearing UKA resulted in a worse ROM than HTO. In addition, there were no significant differences in revision to TKA, relief of postoperative pain, and walking speed between the 2 groups. However, there were differences among the included studies due to different research types, sample sizes, implant designs, matching criteria, operative techniques, and outcome measurements. These differences might be due to significant between-study heterogeneity which could affect the accuracy of the meta-analysis results. Rodkey 2021^[37]^ had a higher revision rate than other studies while contributing to significant heterogeneity. Therefore, a sensitivity analysis was applied to exclude Rodkey 2021 from the analysis to decrease heterogeneity. The results showed that UKA had a lower revision rate than HTO, which is consistent with the previous study.^[43]^

Traditionally, UKA has been recommended for the older sedentary population, and HTO has been indicated for younger active individuals.^[44]^ However, with improvements in implant design and surgical techniques, the traditional distinction between UKA and HTO in terms of surgical indications is becoming less clear. Medial mobile-bearing UKA also showed excellent results in patients under 60 years of age as well as in patients over 60 years of age.^[45]^ Jacquet et al.^[19]^ reported that HTO offered a statistically meaningful faster return to sports and professional activities. HTO had a greater patient rate capable of performing impact activities (62% for HTO vs. 28% for UKA) and increased scores of sport-related functions 2 years after surgery compared to UKA.^[19]^ Song et al.^[20]^ suggested that long-term survival was similar between HTO and UKA in patients with similar demographic data. A previous meta-analysis suggested that UKA was more appropriate for older patients, while HTO provided a better performance of physical activity for younger patients, due to a shorter rehabilitation period and quicker functional recovery.^[46]^ Similar results were observed in relation to postoperative complications, postoperative knee score, and postoperative revision rates to TKA when comparing the OWHTO and UKA groups.^[47]^ In our study, the pooled data demonstrated that UKA was superior to HTO in terms of complications and postoperative Lysholm and HSS scores. UKA may be more suitable for the elderly than HTO because of its safety and better postoperative function.

Various studies have pointed out that mobile-bearing UKA is different from fixed-bearing UKA in terms of restoring natural knee kinematics and reducing contact stress and wear.^[48–52]^ In our study, subgroup analysis was performed to compare fixed-bearing and mobile-bearing UKA with HTO. Compared to HTO, fixed-bearing UKA had a lower ROM. However, no statistically significant difference in ROM was observed between mobile-bearing UKA and HTO. The advantage of HTO is that the integrity of the knee joint is preserved and the postoperative ROM often depends on the preoperative condition. On the other hand, postoperative ROM after UKA depends on the surgical techniques employed, prosthetic designs, and patient preoperative conditions.^[43]^ For younger active individuals, both mobile-bearing UKA and HTO could be considered as surgical options.

Both HTO and UKA effectively relieved postoperative pain, which is the main factor that affects patient satisfaction. Borjesson et al.^[30]^ reported that patients in both groups improved pain during walking. In a study by Weale et al.,^[32]^ 80% of the UKA group presented with mild or no pain compared to 43% of the osteotomy group. Koh et al.^[21]^ involved 123 HTO and 118 UKA patients, and the change in VAS was 5.2 and 5.8, respectively. Zhao et al.^[18]^ showed that the VAS in both groups decreased significantly at 1, 6, 12, and 24 months after surgery. In addition, the VAS of the UKA group 1 month postoperatively was lower than that of the HTO group by 12.2% (*P* < .05), whereas no differences were found at 3 months, 6 months, and 2 years postoperatively (*P* > .05).^[41]^ The relieving pain effect on UKA patients was similar to that in HTO patients. Remarkably, this was consistent with the result of our meta-analysis.

Walking speed is a reliable functional outcome for determining the treatment results of OA patients.^[30]^ Both HTO and UKA can improve postoperative walking speed. Lind et al.^[53]^ demonstrated that walking speed in patients undergoing HTO was significantly enhanced in the postoperative period and that it was not different from that in healthy individuals. The top walking speed was 2.2 m/s in patients submitted to UKA, which was not significantly different from the healthy controls.^[54]^ Borjesson et al. reported^[30]^ that the walking speed of postoperative 5-year in the HTO and UKA groups increased to 1.13 m/s and 1.19 m/s from 1.07 m/s and 1.07 m/s preoperative, respectively. In the other 3studies, the postoperative walking speed with HTO and UKA increased to a certain degree.^[13,33,34]^ Our meta-analysis revealed no significant differences between the 2 groups. HTO resulted in the normalization of many dynamic knee function variables, such as the external knee flexion moment, knee flexion, and walking speed, by reducing the angle of varus and adduction moments of the operated knee.^[53]^ UKA achieved the same effect by reconstructing the damaged surfaces of the osteoarthritic compartment using a metal/plastic construct.^[44]^

Different scoring systems lead to inconsistency in the results of the function score. Cho et al.^[24]^ stated that the postoperative HSS score was significantly higher in the mobile-bearing UKA group than in the OWHTO group. Koh et al.^[21]^ used the new Knee Society scoring system to evaluate patient satisfaction. The satisfaction level in the UKA group was greater than that in the HTO group for more demanding physical tasks such as leisure/recreation activities.^[21]^ These findings are consistent with the study of Kim et al..^[22]^ Additionally, a better Lysholm score was observed in the UKA group than that in the HTO group.^[25,55]^ However, Jacquet et al.^[19]^ compared 91 HTO patients with 117 UKA patients in relation to the University of California Los Angeles score, Knee Injury and Osteoarthritis Outcome Score, Sports Sub-score, and KSS activity score. All these scores were significantly greater in the HTO group than in the UKA group.^[19]^ Yim et al.^[11]^ did not identify significant differences between UKA and HTO for medial unicompartmental OA in relation to return to recreational activity and short-term clinical results. Our meta-analysis found no differences between the 2 groups using pooled data from the KSS, Tegner, and WOMAC scoring systems. Good results were obtained for both the HTO and UKA groups. A prognostic score for medial unicompartmental knee OA should be established to estimate functional outcomes after the treatment of UKA or HTO.

The compound annual growth rate in the use of UKA from 2001 to 2007 in the United States was +4.7%, while that of HTO was −3.9%.^[56]^ Kawata et al. reported^[57]^ that the proportion of patients who underwent UKA increased from 4.0% in 2007 to 8.1% in 2014 and that of tibial osteotomy increased from 2.6% in 2007 to 5.5% in 2014, according to the Diagnosis Procedure Combination database in Japan. In Sweden, UKA use increased threefold during the early decade of the 21st century, while HTO use halved during this period.^[58]^ Niinimäki et al.,^[59]^ using the Finnish National Hospital Discharge Register, noted a steady 6.8% annual decrease in osteotomies, whereas UKA use increased substantially after Oxford UKA was introduced. The current trend indicates that UKA has become increasingly popular in medial unicompartmental OA patients and orthopedists. Fewer complications, higher function scores, and similar ROM might make mobile-bearing UKA more attractive to patients with medial unicompartmental knee OA.

Surgeon experience may have played a key role in the final results. Previous studies have shown that, with increasing experience, operative factors such as surgery time and estimated blood loss decrease, and patient factors such as postoperative complications and length of hospital stay decrease.^[60,61]^ Junior surgeons had higher rates of complications and surgical site infections than did senior surgeons.^[62]^ Postoperative function after UKA was reduced in supervised junior resident and unsupervised senior resident surgeon groups compared to that in attending surgeons.^[63]^ In the included studies, HTO and UKA might have been completed by surgeons with varying levels of experience, which influenced the evaluation results, and thus affected the final conclusions drawn from our meta-analysis.

In comparison with similar previous meta-analysis,^[15,43,46,47,64–69]^ more new studies were updated (published up to November 30, 2021) and more accurate comparison between HTO and UKA was performed. More importantly, in the subgroup analysis, our study is the first to compare HTO with fixed-bearing UKA and mobile-bearing UKA. An important result of this meta-analysis is that the mobile-bearing UKA was not different from the HTO in ROM, whereas the fixed-bearing UKA had less ROM than HTO. This difference can be attributed to the implant design or/and surgical technique.^[49,70]^

We believe that our meta-analysis has certain limitations that deserve consideration. First, the funnel plot indicated that there may be a certain publication bias in our meta-analysis, which may affect the accuracy of the results. Grey literature and unpublished data should be extracted in future studies. Second, most of the included studies incompletely reported random methods, blind methods, and allocation concealment, which could result in a high risk of bias in implementation and measurement. Third, the lack of standardization of the clinical results in the evaluated articles is another important factor that can make it difficult to compare the results of these studies. The results of these studies can be influenced by the potential presence of statistical bias. Fourth, most of the included studies did not mention the details of the studies. Clinical outcomes were influenced by surgical details such as patient characteristics, rehabilitation program, surgeon experience, UKA types, and HTO techniques. Further details should be disclosed to understand the impact of these confounding factors on the results. Fifth, heterogeneity, which is inevitable in meta-analyses, reduces the credibility of the results. Therefore, we applied subgroup and sensitivity analyses to reduce heterogeneity and improve the reliability of the conclusions. Furthermore, 23 of 29 included studies were retrospective studies, which had a lower quality of evidence compared to RCTs. Reliable conclusions need to be confirmed by multicenter RCTs with large sample sizes.

## 5. Conclusions

Both HTO and UKA for the treatment of medial unicompartmental knee OA can achieve good clinical outcomes in relation to revision to TKA, pain relief, and walking speed. However, UKA is better than HTO in minimizing complications and increasing postoperative Lysholm and the HSS scores. The ROM of mobile-bearing UKA was similar to that of HTO. UKA appears to be more suitable for older patients, and both mobile-bearing UKA and HTO are viable surgical options for active younger individuals. The results presented here should be interpreted cautiously as they may contain some limitations. Further multicenter RCTs with large sample sizes are needed to verify the findings of this meta-analysis.

## Author contributions

LH, YX, and JZ provided ideas for this study and helped draft the manuscript. LH and YX collected data. LH, YX, and GY analyzed the data. LW, GY, WC, SG, and WL helped interpret the data. LH, ZT, and JZ edited and reviewed the manuscript. All the authors have read and approved the final manuscript.

## References

[1] Smith WB Steinberg J Scholtes S. Medial compartment knee osteoarthritis: age-stratified cost-effectiveness of total knee arthroplasty, unicompartmental knee arthroplasty, and high tibial osteotomy. Knee Surg Sports Traumatol Arthrosc. 2017;25:924–33. 26520646 10.1007/s00167-015-3821-3

[2] Ledingham J Regan M Jones A. Radiographic patterns and associations of osteoarthritis of the knee in patients referred to hospital. Ann Rheum Dis. 1993;52:520–6. 8346979 10.1136/ard.52.7.520 PMC1005091

[3] Wise BL Niu J Yang M. Patterns of compartment involvement in tibiofemoral osteoarthritis in men and women and in whites and African Americans. Arthritis Care Res. 2012;64:847–52. 10.1002/acr.21606 PMC3340516 22238208

[4] Chen G Xu B Xie J. Comparison of clinical and biomechanical outcomes between partial fibulectomy and drug conservative treatment for medial knee osteoarthritis. Biomed Res Int. 2019;2019:4575424. 31781616 10.1155/2019/4575424 PMC6875010

[5] D’Ambrosi R da Silva M Moura JLM. Radiographic and clinical evolution of the oxford unicompartmental knee arthroplasty. [published online ahead of print July 16, 2021]. J Knee Surg. doi: 10.1055/s-0041-1731718. 10.1055/s-0041-1731718 34520561

[6] Ji W Luo C Zhan Y. A residual intra-articular varus after medial opening wedge high tibial osteotomy (HTO) for varus osteoarthritis of the knee. Arch Orthop Trauma Surg. 2019;139:743–50. 30673869 10.1007/s00402-018-03104-4

[7] D’Ambrosi R Buda M Nuara A. Patellar height after unicompartmental knee arthroplasty: comparison between fixed and mobile bearing. [published online ahead of print October 20, 2021]. J Knee Surg. doi: 10.1007/s00402-021-04183-6. 10.1007/s00402-021-04183-6 34669039

[8] Tuhanioglu U Ogur HU Seyfettinoglu F. High tibial osteotomy in obese patients: is successful surgery enough for a good outcome? J Clin Orthop Trauma. 2019;10(Suppl 1):S168–73. 31695277 10.1016/j.jcot.2018.09.004 PMC6823675

[9] Jin QH Lee W-G Song E-K. Comparison of long-term survival analysis between open-wedge high tibial osteotomy and unicompartmental knee arthroplasty. J Arthroplasty. 2021;36:1562. 33261999 10.1016/j.arth.2020.11.008

[10] Maxwell R Johnston A Lees D. Knee OUTcome Study: a comparison of the patient perceived outcome between high tibial osteotomy, unicompartmental and total knee arthroplasty for medial compartment osteoarthitis in men under age 55. Orthop J Sports Med. 2017;5(5_suppl5):2325967117S2325960016.

[11] Yim J-H Song E-K Seo H-Y. Comparison of high tibial osteotomy and unicompartmental knee arthroplasty at a minimum follow-up of 3 years. J Arthroplasty. 2013;28:243–7. 22854345 10.1016/j.arth.2012.06.011

[12] Akizuki S Shibakawa A Takizawa T. The long-term outcome of high tibial osteotomy: a ten- to 20-year follow-up. J Bone Joint Surg Br Vol. 2008;90:592. 10.1302/0301-620X.90B5.20386 18450624

[13] Weidenhielm L Svensson OK. Broström LÅ, rudberg U. Change in adduction moment about the knee after high tibial osteotomy and prosthetic replacement in osteoarthrosis of the knee. Clin Biomech. 1992;7:91–6. 10.1016/0268-0033(92)90021-U 23915685

[14] Broughton NS Newman JH Baily RA. Unicompartmental replacement and high tibial osteotomy for osteoarthritis of the knee. A comparative study after 5-10 years’ follow-up. J Bone Joint Surg Br Vol. 1986;68:447. 10.1302/0301-620X.68B3.3733813 3733813

[15] Fu D Li G Chen K. Comparison of high tibial osteotomy and unicompartmental knee arthroplasty in the treatment of unicompartmental osteoarthritis. J Arthroplasty. 2013;28:759–65. 23499409 10.1016/j.arth.2013.02.010

[16] Wells GA Shea B O’Connell D. The Newcastle-Ottawa Scale (NOS) for assessing the quality if nonrandomized studies in meta-analyses. Retrieved July 14, 2022. Available at:http://www.ohri.ca/programs/clinical_epidemiology/oxford.htm.

[17] Higgins JPT Altman DG Gotzsche PC. The cochrane collaboration’s tool for assessing risk of bias in randomised trials. BMJ. 2011;343:d5928–d5928. 22008217 10.1136/bmj.d5928 PMC3196245

[18] Zhao S Zhang J Xu Z. Comparative analysis of the medium-term effect of the medial unicompartmental arthroplasty through medial approach next to patellar and high tibial osteotomy on medial compartment osteoarthritis of the knee. Biomed Res-India. 2017;28:3276–80.

[19] Jacquet C Gulagaci F Schmidt A. Opening wedge high tibial osteotomy allows better outcomes than unicompartmental knee arthroplasty in patients expecting to return to impact sports. Knee Surg Sports Traumatol Arthrosc. 2020;28:3849–57. 32008058 10.1007/s00167-020-05857-1

[20] Song SJ Bae DK Kim KI. Long-term survival is similar between closed-wedge high tibial osteotomy and unicompartmental knee arthroplasty in patients with similar demographics. Knee Surg Sports Traumatol Arthrosc. 2019;27:1310–9. 30719541 10.1007/s00167-019-05390-w

[21] Koh IJ Kim MS Sohn S. Predictive factors for satisfaction after contemporary unicompartmental knee arthroplasty and high tibial osteotomy in isolated medial femorotibial osteoarthritis. Orthop Traumatol Surg Res. 2019;105:77–83. 30509622 10.1016/j.otsr.2018.11.001

[22] Kim MS Koh IJ Sohn S. Unicompartmental knee arthroplasty is superior to high tibial osteotomy in post-operative recovery and participation in recreational and sports activities. Int Orthop. 2019;43:2493–501. 30565177 10.1007/s00264-018-4272-5

[23] Ryu SM Park JW Na HD. High tibial osteotomy versus unicompartmental knee arthroplasty for medial compartment arthrosis with kissing lesions in relatively young patients. Knee Surg Relat Res. 2018;30:17–22. 29298462 10.5792/ksrr.17.006 PMC5853178

[24] Cho W-J Kim J-M Kim W-K. Mobile-bearing unicompartmental knee arthroplasty in old-aged patients demonstrates superior short-term clinical outcomes to open-wedge high tibial osteotomy in middle-aged patients with advanced isolated medial osteoarthritis. Int Orthop. 2018;42:2357–63. 29569140 10.1007/s00264-018-3880-4

[25] Krych AJ Reardon P Sousa P. Unicompartmental knee arthroplasty provides higher activity and durability than valgus-producing proximal tibial osteotomy at 5 to 7 years. J Bone Joint Surg. 2017;99:113–22. 28099301 10.2106/JBJS.15.01031

[26] Jeon YS Ahn CH Kim M-K. Comparison of HTO with articular cartilage surgery and UKA in unicompartmental OA. J Orthop Surg. 2017;25:230949901668409. 10.1177/2309499016684092 28176602

[27] Petersen W Metzlaff S. Open wedge high tibial osteotomy (HTO) versus mobile bearing unicondylar medial joint replacement: five years results. Arch Orthop Trauma Surg. 2016;136:983–9. 27154579 10.1007/s00402-016-2465-1

[28] Tuncay I Bilsel K Elmadag M. Evaluation of mobile bearing unicompartmental knee arthroplasty, opening wedge, and dome-type high tibial osteotomies for knee arthritis. Acta Orthop Traumatol Turc. 2015;49:280–7. 26200407 10.3944/AOTT.2015.14.0320

[29] Takeuchi R Umemoto Y Aratake M. A mid term comparison of open wedge high tibial osteotomy vs unicompartmental knee arthroplasty for medial compartment osteoarthritis of the knee. J Orthop Surg Res. 2010;5:65. 20799991 10.1186/1749-799X-5-65 PMC2940897

[30] Börjesson M Weidenhielm L Mattsson E. Gait and clinical measurements in patients with knee osteoarthritis after surgery: a prospective 5-year follow-up study. Knee. 2005;12:121–7. 15749447 10.1016/j.knee.2004.04.002

[31] Stukenborg-Colsman C Wirth CJ Lazovic D. High tibial osteotomy versus unicompartmental joint replacement in unicompartmental knee joint osteoarthritis:: 7–10-year follow-up prospective randomised study. Knee. 2001;8:187–94. 11706726 10.1016/s0968-0160(01)00097-7

[32] Weale AE Newman JH. Unicompartmental arthroplasty and high tibial osteotomy for osteoarthrosis of the knee. A comparative study with a 12- to 17-year follow-up period. Clin Orthop Relat Res. 1994;302:134–7. 8168290

[33] Ivarsson I Gillquist J. Rehabilitation after high tibial osteotomy and unicompartmental arthroplasty. A comparative study. Clin Orthop Relat Res. 1991;266:139–44. 2019043

[34] Jefferson RJ Whittle MW. Biomechanical assessment of unicompartmental knee arthroplasty, total condylar arthroplasty and tibial osteotom. Clin Biomech. 1989;4:232–42.

[35] Hou Y Wang S Wang A. Effect of unicompartmental knee arthroplasty and high tibial osteotomy with tomofix internal fixation in the treatment of unicompartmental knee osteoarthritis. Orthop J Sports Med. 2020;8(9 SUPPL 7):2325967120S0054.

[36] Lin X Liu W Xu X. Unicompartmental knee arthroplasty superior to open-wedge high tibial osteotomy: differences of mechanical parameters and knee function. Chin J Tissue Eng Res. 2021;25:4793–8.

[37] Rodkey DL McMillan LJ Slaven SE. Unicompartmental knee arthroplasty: more conversions, fewer complications than proximal tibial osteotomy in a young population. J Arthroplasty. 2021;36:3878–82. 34481695 10.1016/j.arth.2021.08.001

[38] Chen R Ge H Chen W. Comparison of one-year follow-up effectiveness between high tibial osteotomy and unicompartmental knee arthroplasty for treating medial compartment osteoarthritis of the knee. Chin J Tissue Eng Res. 2020;23:3143–7.

[39] Liu S Zhou G Chen X. Changes in kinematic parameters after unicompartmental knee arthroplasty and high tibial osteotomy. Chin J Tissue Eng Res. 2021;26:406–12.

[40] Watanabe S Akagi R Ninomiya T. Comparison of joint awareness after medial unicompartmental knee arthroplasty and high tibial osteotomy: a retrospective multicenter study. Arch Orthop Trauma Surg. 2021;142:1133–40. 34269892 10.1007/s00402-021-03994-x

[41] Zhang Z Mei Y Zhang L. Therapeutic effects comparison and revision case analysis of unicompartmental knee arthroplasty and open wedge high tibial osteotomy in treating medial knee osteoarthritis in patients under 60 years: a 2-6-year follow-up study. Orthop Surg. 2020;12:1635–43. 32893481 10.1111/os.12761 PMC7767766

[42] Wewers ME Lowe NK. A critical review of visual analogue scales in the measurement of clinical phenomena. Res Nurs Health. 1990;13:227–36. 2197679 10.1002/nur.4770130405

[43] Cao Z Mai X Wang J. Unicompartmental knee arthroplasty vs high tibial osteotomy for knee osteoarthritis: a systematic review and meta-analysis. J Arthroplasty. 2018;33:952–9. 29203354 10.1016/j.arth.2017.10.025

[44] Lobenhoffer P. Indication for unicompartmental knee replacement versus osteotomy around the knee. J Knee Surg. 2017;30:769–73. 28841732 10.1055/s-0037-1605558

[45] Price AJ Dodd CAF Svard UGC. Oxford medial unicompartmental knee arthroplasty in patients younger and older than 60 years of age. J Bone Joint Surg Br. 2005;87-B:1488–92. 10.1302/0301-620X.87B11.16324 16260664

[46] Santoso MB Wu L. Unicompartmental knee arthroplasty, is it superior to high tibial osteotomy in treating unicompartmental osteoarthritis? A meta-analysis and systemic review. J Orthop Surg Res. 2017;12:50. 28351371 10.1186/s13018-017-0552-9 PMC5371236

[47] Huang MQ Li YB Liao CL. Open-wedge high tibial osteotomy and unicomartmental knee arthroplasty in treating medial compartment osteoarthritis of the knee: a meta analysis. Zhongguo Gu Shang. 2019;32:428–33. 31248237 10.3969/j.issn.1003-0034.2019.05.008

[48] Kwon O-R Kang K-T Son J. Biomechanical comparison of fixed- and mobile-bearing for unicomparmental knee arthroplasty using finite element analysis. J Orthop Res. 2014;32:338–45. 24122942 10.1002/jor.22499

[49] Emerson RH Hansborough T Reitman RD. Comparison of a mobile with a fixed-bearing unicompartmental knee implant. Clin Orthop Relat Res. 2002;404:62–70. 10.1097/00003086-200211000-00011 12439239

[50] Whittaker J-P Naudie DDR McAuley JP. does bearing design influence midterm survivorship of unicompartmental arthroplasty? Clin Orthop Relat Res. 2010;468:73–81. 19597898 10.1007/s11999-009-0975-7 PMC2795843

[51] Bonutti PM Dethmers DA. Contemporary unicompartmental knee arthroplasty. J Arthroplasty. 2008;23:24–7. 18922370 10.1016/j.arth.2008.06.025

[52] Burton A Williams S Brockett CL. In vitro comparison of fixed- and mobile meniscal–bearing unicondylar knee arthroplasties. J Arthroplasty. 2012;27:1452–9. 22503333 10.1016/j.arth.2012.02.011

[53] Lind M McClelland J Wittwer JE. Gait analysis of walking before and after medial opening wedge high tibial osteotomy. Knee Surg Sports Traumatol Arthrosc. 2013;21:74–81. 21484389 10.1007/s00167-011-1496-y

[54] Jones GG Kotti M Wiik AV. Gait comparison of unicompartmental and total knee arthroplasties with healthy controls. Bone Joint J. 2016;98-B(10_Supple_B):16–21. 27694511 10.1302/0301-620X.98B10.BJJ.2016.0473.R1 PMC5047137

[55] Krych AJ Reardon P Sousa P. Unicompartmental knee arthroplasty provides higher activity and durability than valgus-producing proximal tibial osteotomy at 5 to 7 years. J Bone Joint Surg Am Vol. 2017;99:113–22. 10.2106/JBJS.15.01031 28099301

[56] Nwachukwu BU McCormick FM Schairer WW. unicompartmental knee arthroplasty versus high tibial osteotomy: united states practice patterns for the surgical treatment of unicompartmental arthritis. J Arthroplasty. 2014;29:1586–9. 24814891 10.1016/j.arth.2014.04.002

[57] Kawata M Sasabuchi Y Inui H. Annual trends in knee arthroplasty and tibial osteotomy: analysis of a national database in Japan. Knee. 2017;24:1198–205. 28797877 10.1016/j.knee.2017.06.005

[58] W-Dahl A Robertsson O Lidgren L. Surgery for knee osteoarthritis in younger patients. Acta Orthop. 2010;81:161–4. 19968599 10.3109/17453670903413186 PMC2852150

[59] Niinimäki TT Eskelinen A Ohtonen P. Incidence of osteotomies around the knee for the treatment of knee osteoarthritis: a 22-year population-based study. Int Orthop. 2012;36:1399–402. 22354471 10.1007/s00264-012-1508-7 PMC3385883

[60] Park Y Lee SB Seok SO. Perioperative surgical complications and learning curve associated with minimally invasive transforaminal lumbar interbody fusion: a single-institute experience. Clin Orthop Surg. 2015;7:91–6. 25729524 10.4055/cios.2015.7.1.91 PMC4329539

[61] Hyun SJ Han S Kim KJ. Adolescent idiopathic scoliosis surgery by a neurosurgeon: learning curve for neurosurgeons. World Neurosurg. 2018;110:e129–34. 29107722 10.1016/j.wneu.2017.10.109

[62] Skovrlj B Cho SK Caridi JM. Association between surgeon experience and complication rates in adult scoliosis surgery: a review of 5117 cases from the scoliosis research society database 2004-2007. Spine (Phila Pa 1976). 2015;40:1200–5. 25996540 10.1097/BRS.0000000000000993

[63] Storey R Frampton C Kieser D. Does orthopaedic training compromise the outcome in knee joint arthroplasty? J Surg Educ. 2018;75:1292–8. 29574018 10.1016/j.jsurg.2018.02.011

[64] Fu YW Liu BG Luo J. Meta analysis of unilateral condylar replacement and high tibial osteotomy in the treatment of medial compartment osteoarthritis of the knee. Zhongguo Gu Shang. 2018;31:1156–63. 30583659 10.3969/j.issn.1003-0034.2018.12.017

[65] Han S-B Kyung H-S Seo I-W. Better clinical outcomes after unicompartmental knee arthroplasty when comparing with high tibial osteotomy. Med. 2017;96:e9268. 10.1097/MD.0000000000009268 PMC5815788 29390376

[66] Rodriguez-Merchan EC. Unicompartmental Knee Osteoarthritis (UKOA): unicompartmental Knee Arthroplasty (UKA) or High Tibial Osteotomy (HTO)? Arch Bone Jt Surg. 2016;4:307–313. 27847841 PMC5100444

[67] Spahn G Hofmann GO von Engelhardt LV. The impact of a high tibial valgus osteotomy and unicondylar medial arthroplasty on the treatment for knee osteoarthritis: a meta-analysis. Knee Surg Sports Traumatol Arthrosc. 2013;21:96–112. 22076053 10.1007/s00167-011-1751-2

[68] Zhang Q-D Guo W-S Liu Z-H. Meta-analysis of unicompartmental knee arthroplasty versus high tibial osteotomy in the treatment of unicompartmental knee osteoarthritis. Zhong hua yi xue za zhi. 2009;89:2768. 20137600

[69] Gandhi R Ayeni O Davey JR. High tibial osteotomy compared with unicompartmental arthroplasty for the treatment of medial compartment osteoarthritis: a meta-analysis. Curr Orthop Pract. 2009;20:164–9.

[70] Cao Z Niu C Gong C. Comparison of fixed-bearing and mobile-bearing unicompartmental knee arthroplasty: a systematic review and meta-analysis. J Arthroplasty. 2019;34:3114–3123.e3. 31474324 10.1016/j.arth.2019.07.005

